# Lessons for future outbreaks from successful contact tracing systems in Asia

**DOI:** 10.1016/j.lanwpc.2025.101563

**Published:** 2025-05-07

**Authors:** Joanna X.R. Tan, Hitoshi Oshitani, Lam Phung Khanh, Charuttaporn Jitpeera, Luca Ferretti, Alex R. Cook

**Affiliations:** a Saw Swee Hock School of Public Health, National University of Singapore, Singapore; b Tohoku University Graduate School of Medicine, Japan; c Ministry of Health, Thailand; d Pandemic Sciences Institute, University of Oxford, Oxford, United Kingdom

**Keywords:** Contact tracing, Outbreak response, Pandemic preparedness, COVID-19

## Abstract

Countries around the world had utilized contact tracing to support public health responses to curb transmission during the COVID-19 pandemic. In particular, countries in East and Southeast Asia had been effective in their contact tracing responses. To understand their successes, the contact tracing systems of Japan, Thailand, Singapore and Vietnam were comparatively analyzed, including the technical aspects of contact tracing approaches, detection and response structures. Through the comparative analysis, we uncovered the key elements within these successful systems, namely speed, capture and accuracy, designed specific for the countries’ settings. For the system to work efficiently, we found that maintaining the balance across speed, capture and accuracy while adapting to the disease epidemiology and environment was essential. Contact tracing will remain a vital strategy to control the next epidemic with a pandemic potential. The lessons learnt could provide guiding principles to help enhance contact tracing systems and prepare for future outbreaks.

## Introduction

Contact tracing is a public health approach to control outbreaks by identifying cases and tracing their contacts, dating back to the past century.^1^ In the modern era, contact tracing may be necessary to mitigate the spread of emerging pathogens before vaccines become available; it can also be useful for endemic infections with a substantial impact, such as HIV and tuberculosis, and for non-endemic infections such as Ebola and measles. During the COVID-19 pandemic, countries around the world implemented contact tracing, but to varying degrees of success, which could be attributable to the high transmissible nature of COVID-19, combined with operational issues such as tracing and testing delays,^2^ and low levels of isolation and quarantine.^3^ As countries revise their pandemic preparedness plans, it behooves them to compare their approach to contact tracing, and other public health and social measures, against successful case studies.

The first year of COVID-19 was marked by extreme differences in the pandemic's impact in parts of East and Southeast Asia and the rest of the world, where the countries in this region maintained low case counts with stable disease control.^4^ Since SARS in 2003, these Asian countries had continually strengthened their public health core capabilities, encompassing components of detection and response structures, which are essential to identify and respond promptly to public health risks and emergencies.^5^ A key element of Asian countries' responses during the COVID-19 pandemic has been an efficient contact tracing system that curbed transmission, sometimes to negligible levels and for long periods until vaccines became available. Despite their effectiveness, these systems have received less attention than they would have deserved.

Contact tracing guidance in the context of COVID-19 has been shared by WHO to guide implementation.^6^ We identified four Asian countries—Japan in East Asia, and Thailand, Singapore and Vietnam in Southeast Asia— that were successful in mounting rapid nationwide responses incorporating contact tracing for sustained period of time. These countries had different economic development ranging from lower-middle income to high income levels^7^ and had distinct public health approaches during the pandemic. A detailed justification on the selection of these four countries is available in the Supplementary. By extracting the principles from WHO's contact tracing guidance, with additional components relating to detection and response structures that supported contact tracing, we analyzed the detailed measures taken by the four Asian countries. Through these components, we developed a conceptual framework comprising of the key elements that make up a successful contact tracing system. With this comparative analysis, we aim to review the technical aspects of different approaches to provide guidance for countries elsewhere to build up and improve contact tracing systems for future outbreaks.

## Overview of nationwide contact tracing

With the emergence of a novel coronavirus from pneumonia cases in Wuhan, China,^8^ the four countries utilized a general approach, as per the International Health Regulations. Prior to importation, border control measures, risk assessments and health advisories were implemented. Extensive epidemiological investigations were conducted upon detection of imported cases. Other than locally acquired sporadic cases, countries began to detect large clusters, such as Japan's cruise ship cluster at Yokohama,^9^ Thailand's cases with linkage to a boxing stadium and night clubs,^10^ Vietnam's Vinh Phuc community cluster^11^ and Singapore's church clusters.^12^ With the diversity of infected cases and clusters in different localities, contact tracing had to be initiated nationwide.

Nationwide contact tracing was generally guided by disease-based strategies. Japan had targeted disease categories, consisting of five categories and a special “designated infectious disease” category, with the corresponding public health measures under the Infectious Disease Control Law.^13^ Thailand had a “dangerous communicable disease” category, and the declaration enabled contact tracing responses in local areas under the Communicable Diseases Act.^14^ Vietnam had three danger levels for infectious diseases, classified as group A-B-C, which served as the foundation for outbreak responses including surveillance and tracing.^15^ Singapore's Infectious Diseases Act provided powers for disease investigations, including contact tracing, and responses were further enhanced for novel disease with pandemic potential through a color-coded Disease Outbreak Response System Condition according to the severity of risk.^16^ As the nationwide operations began, varied approaches were employed (Table 1), as detailed in the subsequent paragraphs. Panel 1, Panel 2, Panel 3, Panel 4 provide the overview of key efforts within each country.

**Table 1:** Overview of contact tracing approaches in Japan, Thailand, Singapore and Vietnam.

	Contact tracing approaches				
	Operations	Manpower surge capacity	Tracing method	Management of contacts	Nationwide applications
Japan	Decentralized: PHCs	Expanded with public health professionals	Multi-faceted: Direct contacts, Backward Tracing	General: Close contacts for self-isolation and monitoring.	COCOA
Thailand	Decentralized: Local CDCs	Expanded with village health volunteers	Multi-faceted: Direct contacts, Source Case Investigation, Active Case Finding, Asymptomatic Infection Finding	Categorized: High-risk for isolation or home quarantine, Low-risk contacts for self-monitoring.	DDC Care, MorChana, Thai Chana
Singapore	Centralized: Ministry of Health	Expanded with civil servants, airline staff	Multi-faceted: Direct contacts, Source and Cluster Investigations.	Categorized: Close contacts for quarantine at designated quarantine locations, Transient contacts under phone surveillance.	TraceTogether, SafeEntry, SGWorkPass
Vietnam	Decentralized: Local CDCs	Expanded with health professionals, civil servants and other volunteers	Multi-faceted: Direct contacts, Source and Cluster Investigations, Generations	Categorized: F1 contacts for quarantine in hospitals or government-run facilities. F2 contacts for home quarantine.	NCOVI, Bluezone

Panel 1 **Overview of Japan's contact tracing efforts**

Japan is a high-income country, with a population of 126 million in 2020. Japan's notifiable diseases and sentinel surveillance system under the Infectious Disease Control Low comprised of five categories of target diseases. In addition to these five categories, the government can designate a designated infectious diseases requiring specific actions for a limited period. Contact tracing is routinely contacted by PHCs in Japan for the different infectious diseases. These PHCs were governed by the local governments, prefectures and major cities.

PHCs were important public health hubs during the pandemic, and contact tracing was mainly driven by PHCs. The specialized contact tracing workforce in PHCs comprised of public health doctors, nurses and other specialists. Rapid deployment of other public health experts supported the expansion of contact tracing activities. Japan employed retrospective contact tracing technique since the beginning, based on the epidemiological characteristics of COVID-19. Japan also used data from contact tracing to formulate control strategies for COVID-19. Backward contact tracing had identified many clusters (‘superspreading events’) in different settings, which improved the understanding of transmission patterns of COVID-19. Common environment factors for clusters, also known as ‘3Cs’, closed environments, crowded conditions, and close-contact settings was widely used as a public health message to prevent transmission in Japan. The “COCOA” application, launched in Japan, utilized Bluetooth features to notify individuals within close proximities of a case.

Panel 2 **Overview of Thailand's contact tracing efforts**

Thailand is an upper-middle income country, with a population of 72 million in 2020. Thailand's national disease surveillance systems are conducted under the Department of Disease Control and the Ministry of Public Health. This includes the indicator-based surveillance system or Report 506 that monitors the communicable diseases trend and event-based surveillance which gathers outbreak news and abnormal events for rapid response. Thailand has a “dangerous communicable disease” category under the Communicable Diseases Act that provide additional legislative powers for disease control.

During the pandemic, contact tracing in Thailand was performed by the local investigation teams or local CDCs. Thailand's operations were governed by the Department of Disease Control, Ministry of Public Health, and supported by the network of regional health offices, provincial health offices, district health offices, and village health volunteers. Thailand's large contact tracing manpower comprised of more than 1000 local CDCs that were specialized in field investigations and outbreaks, and this was supplemented by more than one million village health volunteers who assisted in primary care for designated households. Thailand conducted a multi-faceted contact tracing approach with direct identification of contacts, source case investigation and active case finding. Thailand launched digital applications to enhance contact tracing. The “DDC Care” assisted in the monitoring of symptoms for (i) patients were tested for COVID-19 at the hospital and considered at high risk for COVID-19 infection, and (ii) the close contacts of confirmed COVID-19 cases. The “MorChana” utilized GPS and Bluetooth to identify individuals in close proximities and assist health officials with contact identification. The “Thai Chana” assisted health officials to trace contacts and to assess if businesses were following proper safety protocols. The Communicable Diseases Act served as the legal backbone for Thailand's contact tracing efforts during the pandemic.

Panel 3 **Overview of Singapore's contact tracing efforts**

Singapore is a high-income country, with a population of 5.7 million in 2020. Singapore has robust national disease surveillance systems conducted under the Ministry of Health, including indicator-based surveillance, event-based surveillance, syndromic surveillance and systematic risk assessments of international threats, which capture legally notifiable diseases, disease clusters and any other diseases of concern. Singapore's Infectious Diseases Act 1976 provides powers for disease investigations. Under the event of novel diseases with pandemic potential, the color-coded Disease Outbreak Response System Condition plan outlined the multisectoral public health responses based on assessed disease severity.

Contact tracing in Singapore was conducted by the Ministry of Health during the pandemic. Singapore's contact tracing was conducted by the Ministry of Health, comprising of officers specialized in the control of infectious diseases, and supported with manpower deployment from government personnel and subsequently airline staff. This centralized operation approach allowed coordination and communication between ground contact tracers and policy makers, enabling good situational awareness and adaptability of policies. Singapore employed a multi-faceted approach for contact tracing, including the identification of contacts, potential epidemiological linkages and clusters. Singapore launched “TraceTogether” to assist in contact identification via Bluetooth features, and “SafeEntry” with check-in and check-out mechanisms. Portable devices were issued to the population, enabling access for individuals without digital devices. Both the Infectious Diseases Act, and the COVID-19 Temporary Measures Act that was subsequently enact during the pandemic, provided strong legislative support for investigation and disease control.

Panel 4 **Overview of Vietnam's contact tracing efforts**

Vietnam is a lower-middle income country, with a population of 98 million in 2020. Vietnam has established indicator-based surveillance that includes sentinel surveillance systems for prioritized diseases and the national notifiable disease reporting system, and event-based surveillance system that monitors unusual events. Vietnam classified infectious diseases with three danger levels, namely group A-B-C, that guided outbreak response activities.

During the pandemic, contact tracing activities in Vietnam were coordinated by the local CDCs, with support from local and National Steering Committees for COVID-19 Prevention and Controls. Vietnam's manpower expanded rapidly with civil servants, health professionals, military and student volunteers. Vietnam utilised a multi-faceted contact tracing approach and had a unique contact tracing process to identify F1 (the close contacts of F0) and F2 (the close contacts of F1). Vietnam launched the “NCOVI” to enable users to provide personal information and health declarations, and then utilize the reported information to map hotspots of new infections, and “Bluezone” which identified close contacts via Bluetooth. High uptake rates were achieved. Vietnam's law on prevention and control of infectious diseases, No. 3/2007/QH12, provided powers for stronger leadership and collaboration across government sectors. Pandemic prevention and control was headed by the National Steering Committee for COVID-19 prevention and control, which was also established through legislation.

## Contact tracing approaches

### Operations structure and manpower

With extensive localized COVID-19 transmission, countries were challenged to balance between central coordination and localized outreach. Singapore, a city state, adopted a highly centralized structure, with operations conducted by contact tracing centers in the Ministry of Health.^17^ The centralization enabled strong coordination and communication between tracers and policy makers, complemented with good data security and accountability. The three larger countries took a decentralized approach that leveraged on existing surveillance structures, with support from the central coordinating body. Thailand and Vietnam had local centers for disease control (CDCs) as the main operating centers for contact tracing. Thailand's operations were governed by the Department of Disease Control, Ministry of Public Health, and supported by the network of regional health offices, provincial health offices, district health offices, and village health volunteers.^17^ Vietnam's operation was guided by the local and National Steering Committees for COVID-19 Prevention and Controls, with community support from health professionals, civil servants and volunteers.^18^^,^^19^ Similarly, Japan's contact tracing operations were mainly driven by public health professionals within public health centers (PHCs), and these PHCs were governed by the local governments, prefectures and major cities. The Japan central government role was limited to the issuance of guidance, and data was not shared with the central government. PHCs had other public health services such as distribution of medical resources,^20^ and served as important hubs during the pandemic. These decentralized structures, built on existing surveillance networks, implied lowered startup costs.

Manpower was the next challenge. The contact tracing workforce need to have adequate size and capabilities for all transmission scenarios,^6^ and contact tracers would need to perform tracing efficiently while communicating well with the community. Japan focused on maintaining a specialized contact tracing workforce. PHCs had conducted contact tracing for infectious diseases such as measles, tuberculosis and enterohemorrhagic *Escherichia coli* prior to the pandemic.^21^, ^22^, ^23^ The main workforce had 469 PHCs comprising of public health doctors (>700), public health nurses (>8000), and other public health specialists. Operations were supported by rapid deployment of public health experts from other regions and outside PHCs.^20^ Thailand and Vietnam expanded their workforce through recruiting from the community. Thailand had a sizable contact tracing manpower base, with more than 1000 local CDCs comprising of experts trained in outbreak response. Manpower was supported by more than one million village health volunteers, who had been actively involved in primary health care for assigned households.^24^ Vietnam's tracing workforce was made up of local CDCs, that were trained on COVID-19 specific epidemiological actions upon declaration of the pandemic in Vietnam. Manpower expanded with civil servants, health professions, military and volunteers from healthcare students.^18^^,^^19^ However, managing the rapid expansion was challenging, as Vietnam faced difficulties in training and communication, leading to operational issues ranging from ineffective coordination to unstandardized data collection.^19^ Singapore had manpower deployment plans from past outbreak experiences. The main contact tracing workforce initially comprised of public health officers in the Ministry of Health, enhanced by initial deployment of government personnel and subsequently the deployment of airline staff.^25^^,^^26^ Singapore allocated manpower efficiently through structured teams with specialized roles such as activity mappers and executive officers. This configuration enabled supervised on-the-job learning, with cross-coverage for surge capacities.

### Tracing method

Contact tracing was conducted in many countries using the forward contact tracing approach during the pandemic.^27^^,^^28^ This approach had been effective in containing diseases including Severe Acute Respiratory Syndrome^29^ and Ebola virus disease.^30^ However, for COVID-19, with the characteristic of heterogeneity of secondary transmission, forward tracing would not be as effective if many infected individuals did not generate secondary cases,^31^ and with presymptomatic transmission,^32^ secondary transmission might have occurred prior to case identification. Japan, Thailand, Singapore and Vietnam employed a multi-faceted contact tracing approach for COVID-19. Firstly, in all four countries, the contacts of a confirmed case were traced. Contact identification was carried out using risk-settings approach in Thailand and Singapore.^25^^,^^33^ Through a detailed case interview of the past 14 days movement history and other related information sources, contacts from different settings were identified, including household, workplace, healthcare, travel, and other defined community settings. Procedures were adapted to trace contacts of more complex cases in Singapore, such as foreign worker dormitories and cruise ships. Secondly, tracing methods were developed to identify the source of infection and potential clusters. Japan employed the backward or retrospective contact tracing to identify additional cases of COVID-19. In backward contact tracing, the past activities of multiple infected people were investigated to identify the common source of infection and contacts associated with the source.^34^ The effectiveness of this backward contact tracing method has been supported by epidemiological data from Japan^35^ and Belgium.^36^ Similarly, Thailand conducted source case investigation to find potential source of infection, active case finding approach to investigate the entire community that case resided in, and asymptomatic infection finding in areas with sustained COVID-19 transmission for 28 days or more.^33^ Singapore had parallel approaches: potential links were identified between cases from past movement histories, localities were investigated to find the source and other infected cases, and clusters with ongoing transmission were monitored. Vietnam employed backward contact tracing for up to 14 days prior to the case's disease onset.^37^ Lastly, multiple generations of contacts were traced. This method was unique to Vietnam, with the aim to capture two degrees of contacts to further curb transmission. Once the index case (F0) was detected, the first-degree contacts (F1) and potential source of infection were traced, followed by tracing of F1's contacts (F2).^37^

### Information sources

**Fig. 1:**
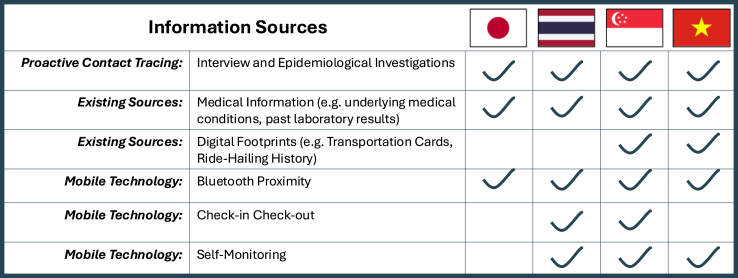
**Summary of information sources.** Proactive contact tracing, including interviews and epidemiological investigations, was conducted in Japan, Thailand, Singapore and Vietnam and served as the main information source to trace the contacts of confirmed cases. Existing information sources also supplemented contact tracing, such as medical records and digital footprints, which could be assessed through an integrated database platform, reporting channels or direct requests for information. Information captured from mobile technology, including Bluetooth proximity indicators, self-monitoring tools ranging from symptomatic monitoring, health declaration to fit-for-work status and check-in check-out digital mechanisms to monitor point of entry into premises also supported the contact tracing processes.

Contact tracing was an active and labor-intensive approach to capture all contacts in close proximity with the case within a defined period. The main information sources were from direct interviews and epidemiological investigations, which were limited by recall bias and reluctance to provide information. Background information sources were used to increase the accuracy of contact tracing.^18^^,^^25^^,^^33^^,^^34^ Information from existing sources included medical records and digital footprints (Fig. 1). Epidemic control via mobile technologies could speed up contact tracing,^3^ and Japan, Thailand, Singapore and Vietnam had developed and implemented digital mobile technologies at nationwide level in early 2020s. Bluetooth technologies assisted exposure identification via proximity indicators: Thailand's MorChana,^38^ Singapore's TraceTogether^39^ and Japan's COCOA^40^ applications utilized Bluetooth features to notify users if they had direct contact with a confirmed COVID-19 case, while Vietnam's Bluezone application warned the contacts of confirmed cases (F1) and the contacts of contacts (F2). Digital technologies with check-in and check-out mechanisms supported contact tracing coverage, including Thailand's Thai Chana which also assisted in surveillance of movement into businesses as part of social distancing measures^41^ and Singapore's SafeEntry, although the latter was hard to integrate into contact tracing efforts due to the often large numbers of other individuals entering or exiting large complexes at the same time. Self-monitoring tools, such as Thailand's DDC Care assisted in symptomatic monitoring of close contacts and high-risk individuals, Vietnam's NCOVI encouraged health declarations,^42^ and Singapore's SGWorkPass combined backend data including test results and quarantine status to provide fit-for-work status for the construction sector.^43^ High population uptake of these digital mobile technologies, however, was difficult to achieve. In Thailand, the acceptance of applications was influenced by elements such as perceived seriousness of illness and simplicity of application navigation.^38^^,^^41^ Japan's COCOA application was impacted by delayed resolution of glitches, and combined with privacy concerns and lack of incentives, led to population usage rate of below 25%.^40^ To encourage voluntary use, Singapore and Vietnam had proactive communications for the digital applications.^39^^,^^42^ Singapore also issued portable TraceTogether devices to the population, enabling access for groups without digital devices.^44^ As such, Vietnam's Bluezone achieved high participation rates of over 30 million downloads while Singapore had an estimate of 92% of population on board the TraceTogether programme.^42^^,^^45^

### Management of cases and contacts

The four countries isolated cases and utilized different approaches for management of identified contacts to prevent further transmission. Japan, Thailand, Singapore and Vietnam isolated and treated cases within healthcare facilities, and the capacities of these facilities expanded rapidly to cope with surge in cases, including expansion of field hospitals in Thailand and Vietnam,^37^^,^^46^ setting up of community care facilities for milder cases in Singapore^47^ and setting up of designated isolation facilities such as hotels designed for the management of mild cases in Japan.^34^ For management of contacts, Japan provided advisories for the identified contacts, where close contacts were advised to self-isolate, and to be tested if symptomatic.^48^ Thailand, Singapore and Vietnam had categorized measures for contacts based on exposure. In Thailand, high-risk contacts were either admitted and tested if they fit “person-under-investigation” or received home quarantine and testing, while low-risk contacts were advised on self-monitoring measures.^33^ In Singapore, close contacts were legally required to quarantine at designated quarantine facilities,^49^ while transient contacts received phone surveillance and testing. In Vietnam, close contacts of the confirmed case (F1) were mandated to quarantine in hospitals or centralized quarantine facilities, and close contacts of F1 (F2) were mandated to quarantine at home.^37^ Quarantine facilities in Vietnam were expanded quickly from hospitals, government-run facilities including dormitories and military barracks, to private facilities such as hotels.

## Detection

### Existing surveillance structures

Contact tracing operations relied on the speed of information flow from existing surveillance channels. Japan, Thailand, Singapore and Vietnam had in place early warning systems to detect potential threats, enabling preparation of response prior to disease importation. These included established networks for international and regional communications^50^ and assessment of international public health threats.^51^^,^^52^ Established reporting networks included event-based surveillance that monitored outbreak and abnormal events reported by health care facilities, laboratories and communities, and indicator-based surveillance that monitored a list of targeted infectious disease with established reporting networks from healthcare institutions and laboratories to the central authority.^51^^,^^53^, ^54^, ^55^ The indicator-based surveillance was supported by legislation, including Japan's Infectious Disease Control Act, Vietnam's Circular No. 54/2015, Thailand's Communicable Diseases Act B.E.2558 (2015) and Singapore's Infectious Diseases Act 1976. During the pandemic, COVID-19 was added as a legally notifiable disease within Japan, Thailand, Singapore and Vietnam. Through these established surveillance networks with mandatory COVID-19 reporting, information flow was hastened from diagnosis to contact tracing teams, enabling quick response.

### Enhancing surveillance during the pandemic

Enhanced pandemic surveillance intended to capture the infected and potentially infected individuals, and generally comprised of indicator-based surveillance and surveillance of targeted populations, in addition to contact tracing. Indicator-based surveillance, relying on medical diagnosis and testing, were guided by case definitions. Confirmed cases, defined by laboratory confirmation of infection, were consistent and similar to the WHO's guidance, enabling case counts to be consistent throughout the pandemic. In contrast, the definition for suspected cases or persons-under-investigation evolved over time. At the start of pandemic when the focus was on importation of cases, suspect cases definitions focused on contact within or history to high-risk countries. The definitions were revised to target risk groups upon community transmission. Japan focused on criteria for vulnerable and non-vulnerable persons.^56^ Thailand focused on risk factors, including point of entry, symptomatic with epidemiological links, healthcare workers and clusters.^33^ Singapore revised the suspect case definition with specific targeted risk factors depending on the local situation.^57^ Vietnam monitored the potential suspect cases from a list of active outbreak areas that was compiled daily.^58^ Surveillance of targeted populations was implemented to identify potential infection among high-risk groups. Border measures were detected and isolated potentially infected individuals arriving at these four countries. Community-based targeted population testing was also implemented. Vietnam had mass testing strategies that adapted over time, including testing at high-risk areas, outbreak areas and multiple testing campaigns.^18^ Singapore implemented targeted mass testing for high-risk groups during outbreak periods such as migrant worker dormitories, and rostered routing testing for workers in high-risk settings including workers in the construction, marine and process sectors and healthcare workers.^59^

## Response

### Legislation for contact tracing

Legislation governed and hence determined the efficiency of operations. Thailand, Vietnam and Singapore had tracing operations supported by legislation. Thailand's Communicable Diseases Act B.E. 2558 (2015) empowered health authorities to collect, analyze, and share data related to infectious diseases for public health purposes, including contact tracing.^14^ Additionally, the Emergency Decree on Public Administration in Emergency Situations enacted in response to COVID-19 provided authorities with enhanced powers to implement containment measures, including quarantine and isolation orders.^60^ In Singapore, the Infectious Diseases Act in Singapore was enacted in 1976 and supported infectious diseases prevention and control. An amendment to the act, which commenced on 25 March 2019,^49^ provided greater legislative support for outbreak control. With the arrival of pandemic in early 2020, the health authorities had legal powers to support nationwide tracing, isolation and quarantine. The COVID-19 Temporary Measures Act further enhanced the powers for control of COVID-19 disease in Singapore.^61^ Vietnam's law on prevention and control of infectious diseases, i.e. No. 3/2007/QH12, strengthened leadership through stipulating the responsibilities within and coordination between governmental bodies.^62^ On the foundation of the law, the National Steering Committee for COVID-19 prevention and control was established quickly, and mobilized pandemic prevention and control activities, including contact tracing. Japan amended the Novel Influenza Act in March 2020 to include COVID-19, enabling legal support for COVID-19 related measures, including declaring state of emergency.^63^ However, there was no law for contact tracing operations, and hence obtaining accurate information was not always possible because some infected persons were reluctant to disclose such information. The use of digital footprint was also not allowed to protect personal information in Japan. As such, conducting backward contact tracing was labor-intensive and time-consuming, and became unfeasible as the number of cases increases.

### Public communications

Communications with public on contact tracing was essential for understanding and building trust and compliance with tracing procedures. The four countries had transparent public communication of the COVID-19 situation, including press release, health advisories and daily COVID-19 situations. This enhanced public's general knowledge of the disease and related measures, including contact tracing. For the identified cases and their contacts, the contact tracers would need to communicate clearly on the investigation process and related health advice. However, there were various on-the-ground challenges amidst the pandemic. Other than contact tracing processes, personnel would need to manage heightened emotions, such as uncertainty and frustration that cases and contacts had about COVID-19 infection, which affected the cooperation with tracers. In Thailand, the public stigma towards patients, and even extended to their families and close contacts, made patients afraid of revealing their risk activities.^64^ Similarly in Vietnam, cooperation with tracers was reduced due to lack of understanding about disease risk, contact tracing procedures, and fear of harmful consequences.^19^ Experiences with scam calls posing as government officials in Singapore impeded contact tracing as individuals were less willing to provide information. Although validation of authenticity could be requested, this additional step slowed down the contact tracing process. Contact tracing would also need to connect with all groups within the country, and population subgroups may have different communication needs. In Thailand, language barriers had been emphasized as barriers to communicating with imported cases during the early period and migrant worker populations.^65^ Social inequalities within the community could also be uncovered during the process of contact tracing. In Singapore, tracers could connect to other agencies such as the People's Association to provide additional community support for cases and contacts,^66^ outside of the typical contact tracing operations.

## Elements of a successful contact tracing system

**Fig. 2:**
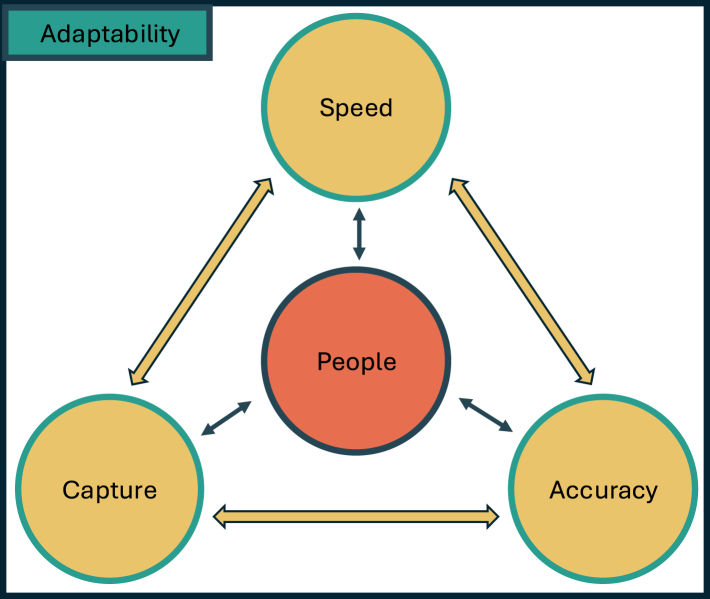
**Elements of a successful contact tracing system.** The common key elements within the contact tracing systems of Japan, Thailand, Singapore and Vietnam were speed, capture and accuracy. *Speed* was enhanced with efficient teams and established disease surveillance and response structures. Good *capture* curbed disease transmission through multi-faceted contact tracing, enhanced surveillance, isolation and quarantine. High *accuracy* was maintained via good communication and empowerment of contact tracers. People, at the center of a successful system, represented the important of engagement and trust with the population. The arrows portrayed the interaction between the elements, and the importance of maintaining resource balance. The success of the system also relied on adaptability of the system to the environment and disease epidemiology.

Comparing the key structures, we analyzed both the common and disparate aspects that led to successful contact tracing systems within Japan, Thailand, Singapore and Vietnam, and identified the key elements that make up a successful contact tracing system (Fig. 2). These contact tracing systems maintained a balance of speed, capture and accuracy that, to be effective, required the compliance of the population and therefore had to be designed to obtain their broad support. The *speed* of the systems was due to efficient teams through existing manpower structures and fast information flow through established surveillance systems. These systems *captured* groups at risk through multi-faceted contact tracing and enhanced surveillance, isolated the infected and quarantined the potentially infected to prevent further transmission. The systems had well-structured and highly trained teams that were able to communicate effectively with the ground and were empowered by structures such as legislation and access to multiple information sources, enabling contact tracing to operate at high *accuracy*. The support of the people was central to the success of all four systems: there had to be trust to adhere to the measures, sometimes bolstered by legislation to enhance compliance by threatening punishment otherwise. For a successful contact tracing system, these elements of speed, capture and accuracy need to work well together and be continuously adaptable, to safeguard from issues such as delays and low rates of isolation and quarantine. Despite the successes, these countries had encountered challenges balancing resources for the system, especially when cases increase.

In Japan's case, the devolved nature of public health and the emphasis on protection of individual rights meant the government took a relatively soft approach, through public health advisories, which meant heavier reliance on social responsibility rather than legislation and enforcement. More resources had, therefore, to be channeled into increasing compliance, including efforts from tracers, which could impact the speed of tracing and thereby its effectiveness. Moreover, with low uptake of digital application, the accuracy of contact tracing depended mainly on proactive tracing by PHCs. Obtaining accurate information was especially challenging in metropolitan areas such as Tokyo as people from wide geographic areas visited the same place. When cases were few, such as in the first COVID-19 wave, contact tracing was conducted effectively, even in metropolitan cities such as Osaka.^67^ However, with high dependence on PHCs, good speed and accuracy were difficult to maintain under high caseloads, culminating in contact tracing becoming infeasible in the waves caused by the Omicron variant, and contact tracing was only conducted in special settings such as hospitals and long-term care facilities. A lesson from Japan's experience therefore is that having sufficient resources within each element is important to maintain a functioning contact tracing system.

Having high sensitivity of tracing capture meant more resources required for contact tracing. Vietnam implemented an extensive method to capture contacts over two “generations”, which, in combination with good speed, access and popular support, enabled suppression of the outbreak in its early phases. However, this wide capture became difficult to sustain amidst high caseloads as more resources were required for good speed and accuracy. Although the operations expanded, the speed of contact tracing was affected, with ineffective coordination in ground operations. Similarly, contact tracing was impacted with incomplete and inaccurate information, as it became difficult to conduct operations without essential resources such as personal protective equipment and landlines, while lacking proper training of new tracers and inefficient manpower allocation.^19^ Vietnam's approach, which worked well with low number of community cases, would have required infeasible resources to maintain a sensitive capture when the cases increased.

Should the population have subgroups with higher risk of disease transmission, it is essential for contact tracing to capture these groups as well. Singapore experienced large outbreaks in dormitories for blue-collar foreign workers in the first half of 2020.^68^ People living in dormitories had different characteristics from the general population: high living densities, distinct from the rest of the population, and with low socioeconomic status. Singapore as a whole had a good tracing system for the general population, but the system was not sensitive to contain transmission within dormitories. To contain transmission, Singapore accelerated capture through treatment and isolation with setting up of medical posts within dormitories, and targeted dormitory testing strategies.^69^ Contact tracing methods were modified to fit the dormitory structures and worksites. To enhance adherence to measures, a digital “AccessCode” system monitored health status for work resumption.^43^ Through a combination of herd immunity and channeling resources to enhance capture for the subgroup while maintaining good tracing speed, the transmission in dormitories was contained in the late 2020.

## Discussion

The experiences of Japan, Thailand, Singapore and Vietnam highlight the essential elements for contact tracing to be successful, and conversely when it fails. These highly efficient systems enabled rapid implementation of tracing and isolation and was therefore able to prevent secondary and subsequently tertiary infections. This had worked in favor of COVID-19's pathogen characteristics: a shorter implementation time scale relative to the virus generation time. However, this may not always apply to pathogens with a shorter generation time. Similarly, countries that do not have such efficient systems, may not be able to implement effective contact tracing during the pandemic due to this effect. As we look back, COVID-19 had been a unique pandemic and contact tracing had been challenging; the original COVID-19 viral strain had high transmissibility, and the transmissibility continued increasing with subsequent new variants. The conventional wisdom is that contact tracing quickly breaks down during the pandemic due to the sheer volume of cases, however the four countries have managed to sustain their contact tracing efforts for long periods to suppress the rise in cases. Yet, although contact tracing is a vital strategy in the first pandemic stage to reduce transmission and achieve containment, the value decreases as the pandemic stages progress and cases increases, and deciding the timing to stop contact tracing was challenging for governments.

Each country can have their own resource limitations and challenges. While our study may not be the representative sample of countries globally, we believe that the guiding principles from these countries have important implications for how other countries can improve their contact tracing for future pandemics. The countries that were selected in our study, though diverse, had more pro-social and less individualistic cultures. Contact tracing is dependent on people's support, and the successes of such approaches might differ for countries with stronger individualistic cultures. Techniques were discussed to combat the transmission of COVID-19. We believe that these approaches can be replicated for pathogens with similar properties such as generation time and transmission heterogeneity. Our study did not seek to compare the effectiveness of various strategies for speed, capture and accuracy quantitatively, and further research into contact tracing methods, isolation, quarantine and surveillance strategies would be valuable to guide contact tracing policies for more effective and catered responses. The societal aspects of contact tracing, including public stigma, community support and social inequalities, were highlighted as communication challenges for ground operations. Our study did not explore the different perspectives of contact tracing within the community through fieldwork, and future work on the social impacts of contact tracing approaches and acceptability to the population will be essential. Although we have discussed the four countries' contact tracing approaches in terms of COVID-19, they may be applicable to other pathogens with differential successes, and as newer technologies and tools become available, advancing these aspects should be the subject of future studies.

The next novel disease might be different from COVID-19. Contact tracing will continue to be a vital or even the most important strategy to control the next epidemic with a pandemic potential. This is especially so for diseases with high case fatality ratio but potentially feasible containment, such as SARS, where containment should be aimed for immediately rather than waiting for the development of vaccines. With disease threats ever looming, the future of contact tracing systems must be strengthened for the future. The lessons learned from these countries could help enhance public health policies to develop efficient contact tracing operations for future outbreaks.

## Contributors

ARC and JXRT conceived the study. CJ, HO, JXRT and LPK conceptualized and drafted the country-specific contact tracing systems for Thailand, Japan, Singapore and Vietnam respectively. JXRT wrote the first draft of the manuscript, and the manuscript was reviewed and edited by HO, LPK, CJ, LF and ARC.

## Declaration of interests

HO received the COVID-19 research grant from the Ministry of Health, Labor and Welfare. Other authors declare that they have no known competing interests.

## Appendix A Supplementary data

Supplementary Country Selection
