# Supplementary material for: Lessons for future outbreaks from successful contact tracing systems in Asia
Source: Lancet Reg Health West Pac. 2025 May 7;58:101563. doi: 10.1016/j.lanwpc.2025.101563 (PMC12134535; doi:10.1016/j.lanwpc.2025.101563)
Supplement: Supplementary Country Selection [file mmc1.pdf]

Supplementary

Country Selection

Asia has maintained low COVID-19 cases and deaths during the pandemic, as compared to the rest of the world, and thus keeping the impact of the pandemic minimal. As of 31 Dec 2020, the total cumulative number of confirmed COVID-19 cases was 4349 per million people in Asia, as compared to 33396 per million people in the European Union, 57323 per million people in the United States and 10306 per million people in the world (inclusive of Asia) (1). Similarly, the total cumulative confirmed COVID-19 deaths remained low in Asia at 71 per million people as of 31 Dec 2020, as compared to 862 per million people in the European Union, 1031 per million people in the United States and 242 per million people in the world (1).

With this study focused on Asia, we looked at the countries that had conducted contact tracing within Asia. Given that countries could have maintained low COVID-19 case counts due to factors other than contact tracing, we included countries in study using the following criteria:

- **Economic status** by World Bank's income group categories (2). This selection criteria were included to ensure that countries compared were representative of income levels in Asia and could enable better generalizability for countries with varying resource levels.
- **Good COVID-19 control**, indicated by low cumulative confirmed COVID-19 death. Low COVID-19 death was chosen rather than confirmed case counts, as COVID-19 could present as an asymptomatic disease and the testing rates would affect the number of cases detected and notified. Severe illness and subsequent deaths during the pandemic, however, would likely undergo COVID-19 testing as part of international reporting guidelines (3), and would be a more accurate indicator. Time range was from start of pandemic to end of June 2021, where many countries still conducting comprehensive contact tracing (4).
- **Distinct public health approach,** which could provide new insights on contact tracing for other countries.
- **Good public health systems**, indicated by WHO Joint External Evaluation (5). WHO Joint External Evaluation assessed country's preparedness in terms of International Health Regulations implementation, and the overall score would indicate the level of preparedness for good public health responses. Criteria was included as contact tracing is dependent on these preparedness structures.

In total, we selected four Asian countries that were outstanding after considering the combination of income levels, evidence of strong systems, good COVID-19 control and distinct approaches:

Vietnam has maintained a low cumulative confirmed COVID-19 deaths over the pandemic, at 0.87 per million people as of 30 June 2021 (1). Vietnam's joint external evaluation, which was evaluated in 2016, had a higher-than-average score as compared

with the low-middle income countries (i.e. 137 compared to the average of 120.3 in low-middle income countries) (6,7). Vietnam also had a distinct public health approach: the implementation of a comprehensive third-degree contact tracing system, which was unique in the world (8).

Thailand had the lowest COVID-19 deaths among the upper-middle income Asian countries at 28 confirmed cumulative COVID-19 deaths per million people as of 30 June 2021 (1). Thailand's joint external evaluation, evaluated in 2022, show strong capacities with at least a score of 4 for all indicators in 17 out of 19 technical areas (9). Thailand had vast community-based support for contact tracing operations during the pandemic, comprising of trained local disease units and village health volunteers (10).

Singapore had the lowest COVID-19 death as compared to other Asian high-income countries, at 6.4 confirmed cumulative COVID-19 deaths per million people as of 30 June 2021 (1). Singapore's joint external evaluation, evaluated in 2018, was among the top two of the countries evaluated in the same period (11,12). During the pandemic, Singapore successfully launched a digital contact tracing program during the pandemic and achieved high population-wide uptake rates (13).

Japan had 118 confirmed cumulative COVID-19 deaths per million people as of 30 June 2021 (1). Although Japan had a higher-than-average death count as compared to the other high-income Asian countries (though significantly lower than countries from other income levels), Japan was well-known in the other areas: Japan was ranked among the top five countries globally based on joint external evaluation scores (11,14), indicating a strong preparedness system prior to the pandemic. During the pandemic, Japan had a distinct approach by advocating for the implementation of backwards contact tracing, with its effectiveness established through research and used in real-life implementation (15,16).

References

1. Edouard Mathieu, Hannah Ritchie, Lucas Rodés-Guirao, Cameron Appel, Daniel Gavrilov, Charlie Giattino, Joe Hasell, Bobbie Macdonald, Saloni Dattani, Diana Beltekian, Esteban Ortiz-Ospina and Max Roser (2020) - “Coronavirus (COVID-19) Cases” Published online at OurWorldinData.org. Retrieved from: 'https://ourworldindata.org/covid-cases' [Online Resource]

2. World Bank Country and Lending Groups – World Bank Data Help Desk [Internet]. [cited 2025 Mar 11]. Available from: https://datahelpdesk.worldbank.org/knowledgebase/articles/906519-world-bank-country-and-lending-groups

3. World Health Organisation. International Guidelines for Certification and Classification of COVID-19 as Cause of Death [Internet]. 2020. Available from: https://www.who.int/docs/default-source/classification/icd/covid-19/guidelines-cause-of-death-covid-19-20200420-en.pdf

4. Blavatnik School of Government, University of Oxford (2023) – with minor processing by Our World in Data. “Which countries do COVID-19 contact tracing?” [dataset]. Blavatnik School of Government, University of Oxford, “Government Response Tracker (OxCGRT)” [original data]. Retrieved from: https://ourworldindata.org/grapher/covid-contact-tracing

5. World Health Organization. Joint External Evaluation Tool Third Edition [Internet]. 2022 Jun. Available from: https://iris.who.int/bitstream/handle/10665/357087/9789240051980-eng.pdf?sequence=1

6. World Health Organization. Joint external evaluation of IHR core capacities of Viet Nam [Internet]. 2016 [cited 2024 Jul 10]. Available from: https://www.who.int/publications/i/item/WHO-WHE-CPI-2017.21

7. Chiu De Vázquez C, Jou YC, Nyan HHL, Asakura M, Watanabe K, Lowbridge C. Estimating Joint External Evaluation Scores Using Country Data from 77 Countries, 2016-2018. Health Security. 2021 Apr 1;19(2):150–62.

8. Nguyen TV, Tran QD, Phan LT, Vu LN, Truong DTT, Truong HC, et al. In the interest of public safety: rapid response to the COVID-19 epidemic in Vietnam. BMJ Glob Health. 2021 Jan;6(1):e004100.

9. World Health Organization. Joint external evaluation of IHR core capacities of Thailand [Internet]. 2022. Available from: https://www.who.int/publications/i/item/9789240080270

10. Krassanairawiwong T, Suvannit C, Pongpirul K, Tungsanga K. Roles of subdistrict health office personnel and village health volunteers in Thailand during the COVID-19 pandemic. BMJ Case Rep. 2021 Sep 14;14(9):e244765.

11. Traore T, Shanks S, Haider N, Ahmed K, Jain V, Rüegg SR, et al. How prepared is the world? Identifying weaknesses in existing assessment frameworks for global health security through a One Health approach. The Lancet. 2023 Feb 25;401(10377):673–87.

12. World Health Organization. Joint external evaluation of IHR core capacities of Singapore [Internet]. 2018. Available from: https://www.who.int/publications-detail-redirect/WHO-WHE-CPI-REP-2018.25

13. TraceTogether Programme Data Deregistration (PQ Reply by Minister Vivian Balakrishnan) [Internet]. Available from: https://www.smartnation.gov.sg/media-hub/parliament/20210510/

14. World Health Organization. Joint external evaluation of IHR core capacities of Japan [Internet]. Geneva: World Health Organization; 2018. Available from: https://iris.who.int/handle/10665/274355

15. Endo A, Abbott S, Kucharski AJ, Funk S. Estimating the overdispersion in COVID-19 transmission using outbreak sizes outside China. Wellcome Open Res. 2020;5:67.

16. Imamura T, Saito T, Oshitani H. Roles of Public Health Centers and Cluster-Based Approach for COVID-19 Response in Japan. Health Secur. 2021 Apr 1;19(2):229–31.
